# Localizations and functions of septins are susceptible to epitope tagging

**DOI:** 10.1101/2025.05.13.653749

**Published:** 2025-05-14

**Authors:** Jack R. Gregory, Ian Mikale A. Llaneza, Aysha H. Osmani, Haley E. Gosselin, Jian-Qiu Wu

**Affiliations:** a Department of Molecular Genetics, The Ohio State University, Columbus, OH 43210; b Molecular, Cellular, Developmental Biology Graduate Program, The Ohio State University, Columbus, OH 43210; c Cellular, Molecular and Biochemical Sciences Program, The Ohio State University, Columbus, OH 43210; d Ohio State Biochemistry Program, The Ohio State University, Columbus, OH 43210

**Keywords:** anillin Mid2, arrestin Art1, cytokinesis, fission yeast, septin, Spn1, Spn4, *S. pombe*, septum, tdTomato

## Abstract

Septins are hetero-oligomeric cytoskeletal proteins that assemble into filaments and scaffolds on the plasma membrane to aid cytokinesis, morphogenesis, and other processes. Epitope tagging is widely used in studying septin localizations and functions. However, their functionalities are rarely tested rigorously because of technical challenges. Fission yeast provides an ideal genetic system to test functionalities of tagged septins. Septins Spn1 and Spn4 localize exclusively to the division site as double rings during cytokinesis with mEGFP or mYFP tags. However, both septins also localized to puncta or linear structures across the plasma membrane during interphase when tagged with tdTomato. It was proposed that these interphase septin structures are important for the localizations and functions of several proteins including the NDR-kinase Sid2 and active Cdc42. By analyzing cell morphology, cytokinesis/septation defects, and genetic interactions between tagged septins and the arrestin *art1Δ* and anillin *mid2Δ,* we find that septins tagged with tdTomato or 3HA are not functional. Moreover, Sid2 appearance at the division site is later than septins and delayed in septin mutants, contrary to previous report. Our data emphasize the need for rigorous functional tests of tagged septins and caution in interpreting function and localization data because septin polymers are susceptible to perturbations.

## INTRODUCTION

Septins are a class of proteins considered to be the fourth element of the cytoskeleton (Mostowy and Cossart, 2012). These proteins were first discovered in a landmark screen for cell division controlling proteins in the budding yeast *Saccharomyces cerevisiae* (Hartwell, 1971). Further studies in budding yeast discovered a filamentous ring at the bud site and bud neck (Byers and Goetsch, 1976). The pioneer immunofluorescence staining using specific antibodies by the Pringle laboratory revealed that Cdc3, Cdc10, Cdc11, and Cdc12 are necessary for this ring to form, and the four proteins localize to the cell division site and are essential for cytokinesis (Haarer and Pringle, 1987; Ford and Pringle, 1991; Kim et al., 1991). This family of proteins were subsequently named as the septins and was found to exist in nearly all eukaryotic cells except land plants (Neufeld and Rubin, 1994; Kinoshita et al., 1997; Nguyen et al., 2000; Surka et al., 2002; Kwitny et al., 2010; Juvvadi et al., 2011; Nishihama et al., 2011).

Septins are essential for cytokinesis, cell polarization, exocytosis, neuronal development, and many other functions in eukaryotic cells. They act as a barrier to diffusion between the mother cell and the emerging bud in budding yeast (Takizawa et al., 2000) and as a barrier for membrane diffusion in mouse sperm and cilia (Kwitny et al., 2010). The budding yeast septins function as a scaffold to coordinate contractile ring assembly, the synthesis and deposition of chitin in new cell walls (DeMarini et al., 1997), and most other processes at the bud neck (Gladfelter et al., 2001). As sensors of membrane curvature, septins also modulate and compartmentalize the plasma membrane (Bridges et al., 2016). In filamentous fungi and fungal pathogens, septins assume many unique structures within cells , which are important for fungal morphogenesis and infection (Gladfelter, 2006; Harris, 2006). Septins also bind to and stabilize microtubules in human cell lines (Surka et al., 2002). They have been implicated in many vesicle trafficking pathways and interact with the exocyst complex during cytokinesis (Singh et al., 2024). Additionally, septins are enriched in the brain and required for the formation of neurites (Hsu et al., 1998; Radler and Spiliotis, 2022). Considering their numerous functions, it is not surprising that mutations in septins have been implicated in many diseases, including Alzheimer’s, cancers, bipolar disorder, infertility, birth defects, and other neurological disorders (Kinoshita et al., 1998; Pissuti Damalio et al., 2012; Das and Kunwar, 2025). The septins have also been shown to be necessary in combating a plethora of viruses including Hepatitis C, Influenza, and Vaccinia Virus (Khairat et al., 2024). Moreover, the septins of certain fungal species are potentially useful targets for anti-fungal drug development due to their essential role in membrane transport (Eisermann et al., 2023). The diverse roles of septins spanning across many species, their association in human pathology, and their implications in therapeutics make it crucial to coherently understand how the septins function throughout the cell cycle.

Septins are GTP binding proteins, and most cell types express multiple different septins. Septins are capable of binding to one another to form multimeric complexes, often as hexamers or octamers (Garcia et al., 2011; Fung et al., 2014). These multimeric complexes can further polymerize into various higher-order structures such as rings, hourglasses, filaments, and gauzes (Byers and Goetsch, 1976; Rodal et al., 2005; Vrabioiu and Mitchison, 2006; Garcia et al., 2011; Bridges et al., 2014). These septin structures occupy different locations in the cell, depending on their function, including cilia, division sites, microtubules, and fungal infection sites (Bridges and Gladfelter, 2015). The most notable and well-studied location of septins is at the division site, where they are indispensable for cytokinesis (Haarer and Pringle, 1987; Ford and Pringle, 1991; Kim et al., 1991; Longtine et al., 1996; Berlin et al., 2003; Tasto et al., 2003). As seen by the determined structure (Sirajuddin et al., 2007), the septins interact via two faces: the G interfaces and the NC interfaces. These are named after the GTP binding domain in the septins and the interface formed from the available N-terminus and C-terminus. Septins also include the septin unique element (SUE) and the coiled-coil domain, which is commonly within their C-termini (Sirajuddin et al., 2007; Shuman and Momany, 2021). Upstream of the GTP binding domain is the polybasic region which contains many basic amino acids that can interact with phosphatidic acids on the membrane (Bridges and Gladfelter, 2015). Septins have many binding partners (Gladfelter et al., 2001; Joberty et al., 2001; Surka et al., 2002; Singh et al., 2024). The nature of septin polymerization into heteromeric higher-order structures and their numerous binding partners makes them susceptible to perturbation by epitope tags, which are indispensable in their utilities for studying all proteins including septins.

The fission yeast *Schizosaccharomyces pombe* is an attractive, genetically tractable model organism that has been utilized to understand fundamental cellular processes (Balasubramanian et al., 2004; Pollard and Wu, 2010). *S. pombe* has seven septins Spn1-Spn7 (Longtine et al., 1996; Onishi et al., 2010). Spn5-7 only function in sporulation during meiosis (Onishi et al., 2010). Together with Spn2, Spn5-7 form a scaffold, bind to PI(4)P lipid, and guide the expansion of the plasma membrane to envelope the forming spores (Onishi et al., 2010). Spn1, Spn2, Spn3, and Spn4 are expressed during vegetative growth and localize to the division site as double rings at the rim of the division plane (Longtine et al., 1996; Berlin et al., 2003; Tasto et al., 2003; An et al., 2004; Onishi et al., 2010; Wu et al., 2010). Spn1 and Spn4 are more important than Spn2 or Spn3 as without either Spn1 or Spn4, no other septin can localize to the division site (An et al., 2004). Septins are important for septum formation and daughter-cell separation by interacting with the exocyst complex to regulate the secretion of the glucanases (Martin-Cuadrado et al., 2005; Singh et al., 2024). Septin localization at the division site in fission yeast is regulated by the anillin Mid2 during cytokinesis (Berlin et al., 2003; Tasto et al., 2003; Wu et al., 2010). However, there are conflicting reports on how septins function and where they localize during interphase. Earlier studies have shown no specific localization of septins on the plasma membrane during interphase (Berlin et al., 2003; Tasto et al., 2003; Wu et al., 2010). However, several recent studies have shown that Spn1 and Spn4 localize as puncta or linear structures all over the plasma membrane and serve as diffusion barriers for proteins such as the NDR kinase Sid2, active Cdc42 GTPase, the Rho GAPs Rga6 and Rga4 (Zheng et al., 2022; McDuffie et al., 2024). It has been proposed that septin puncta or linear filaments on the plasma membrane inhibit the diffusion of Sid2, an NDR-family kinase in the Separation Initiation Network (SIN) pathway, and prevent Sid2’s premature accumulation at the division site (Zheng et al., 2018). In addition, it has been proposed that septins help restrict active Cdc42 to cell tips during interphase for polarized growth (Zheng et al., 2022; McDuffie et al., 2024).

In this study, we evaluated the localization and function of the septins in fission yeast using Spn1 and Spn4 with different epitope tags. By analyzing the localization of the septins, cell morphology, cytokinesis/septation defects, cell integrity, and genetic interactions between tagged septins and the arrestin *art1Δ* and anillin *mid2Δ,* we show that septins Spn1 and Spn4 tagged with mEGFP and mYFP are functional and localize normally to the division site. By contrast, the septins tagged with 3HA, tdTomato, and other red fluorescent tags are less functional or not at all. This resulted in localization artifacts on the plasma membrane or a loss of septin function. In addition, our data do not support the hypothesis that septins function as diffusion barriers during interphase in fission yeast. Our results emphasize the need to rigorously test the functionality of septins and other proteins with epitope tags. Caution is warranted when interpreting septin functions and localizations because septin polymers are susceptible to various perturbations.

## RESULTS

### The same septin has different localizations with different epitope tags

**Figure 1:**
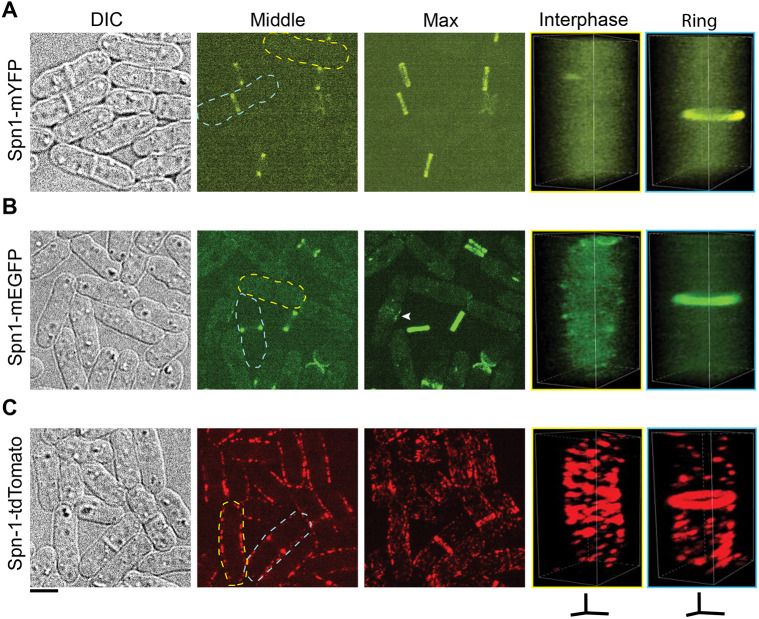
Apparent Spn1 localizations depend on the fluorescent tags. Cell morphology and Spn1 localization in strains expressing Spn1-mYFP (A), Spn1-mEGFP (B), and Spn1-tdTomato (C). Differential Interference Contrast (DIC), fluorescence images at the middle focal plane and the maximal intensity projection of 25 slices with 0.3 μm spacing are shown. 3D volumetric projection of representative interphase and dividing cells are on the right. Scale bar, 5 μm. Axial bars, 2 μm.

To assess the conflicting claims about septin localizations, we analyzed the localizations of the two most important septins in *S. pombe* during vegetative growth using fluorescence confocal microscopy. Septins Spn1 and Spn4, whose deletions have the strongest phenotypes (Berlin et al., 2003; Tasto et al., 2003; An et al., 2004), were tagged with different fluorescent tags at their C-termini and expressed under their native promotors at endogenous chromosomal loci. In cells expressing Spn1-mYFP or Spn1-mEGFP, Spn1 diffused in the cytoplasm without distinct localization on the plasma membrane during most of interphase (Figure 1, A and B). Occasionally, Spn1 formed a few stochastic puncta on the plasma membrane, which were rare. In cells with a septum during cell division, Spn1 localized to non-contractile rings at the division site (Figure 1, A and B), which could be resolved as double rings at higher spatial resolution. Spn1 appeared at the division site just before septum formation as a fuzzy band (Figure 1B, arrowhead), which quickly resolved into septin rings, as the fluorescence intensity in the cytoplasm dramatically decreased (Figure 1, A and B). During daughter-cell separation after septum maturation, Spn1 rapidly spanned the entirety of the new cell ends from the double rings, and then gradually disappeared (Figure 1, A and B). These observations are consistent with previous publications (Berlin et al., 2003; Tasto et al., 2003; An et al., 2004; Wu et al., 2010). In contrast, Spn1-tdTomato localized all over the plasma membrane as puncta or short linear structures in every cell (Figure 1C). In cells with septin rings during cytokinesis and septum formation, Spn1-tdTomato still remained in these structures on the plasma membrane outside the division site, although with reduced fluorescence intensity or structures (Figure 1C). These observations with Spn1-tdTomato are also consistent with some other previous publications (Zheng et al., 2018; Zheng et al., 2022; McDuffie et al., 2024).

**Figure 2:**
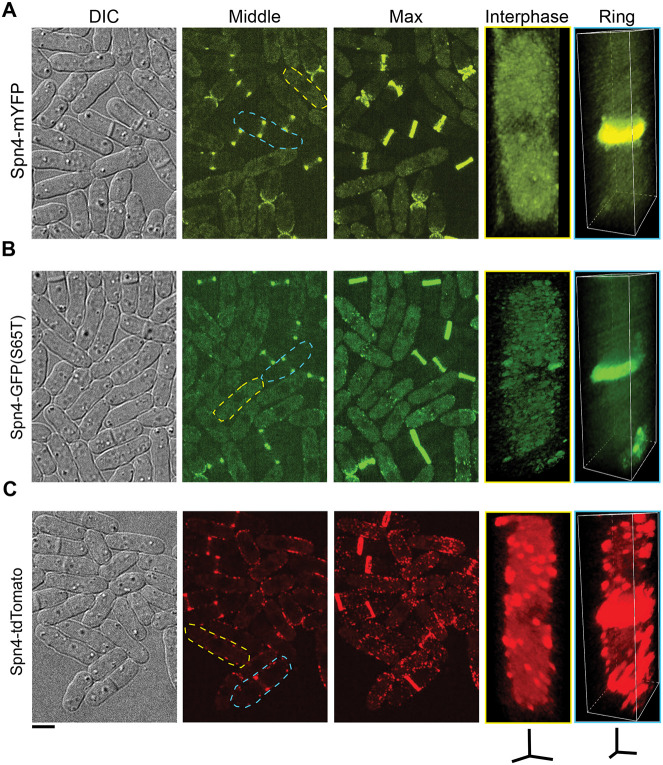
Apparent Spn4 localizations depend on the fluorescent tags. Cell morphology and Spn4 localization in strains expressing Spn4-mYFP (A), Spn4-GFP(S65T) (B), and Spn4-tdTomato (C). DIC, fluorescence images at the middle focal plane and the maximal intensity projection of 25 slices with 0.3 μm spacing are shown. 3D volumetric projection of representative interphase and dividing cells are on the right. Scale bar, 5 μm. Axial bars, 2 μm.

Tagged Spn4 behaved almost identically to Spn1 (Figure 2). Localizations of Spn4-mYFP and Spn4-GFP(S65T) resembled those of Spn1 tagged with mYFP or mEGFP (Figure 2, A and B), although there were slightly more puncta in cells expressing Spn4-GFP(S65T), which could be due to GFP(S65T)’s weak dimerization. (Oltrogge et al., 2014). Spn4-tdTomato localizations were also identical to those of Spn1-tdTomato (Figure 2C). These conflicting data on Spn1 and Spn4 raised questions as to which tagged septins are more functional and which septin localizations are real.

### A flexible linker or smaller tag do not restore septin functionality

**Figure 3:**
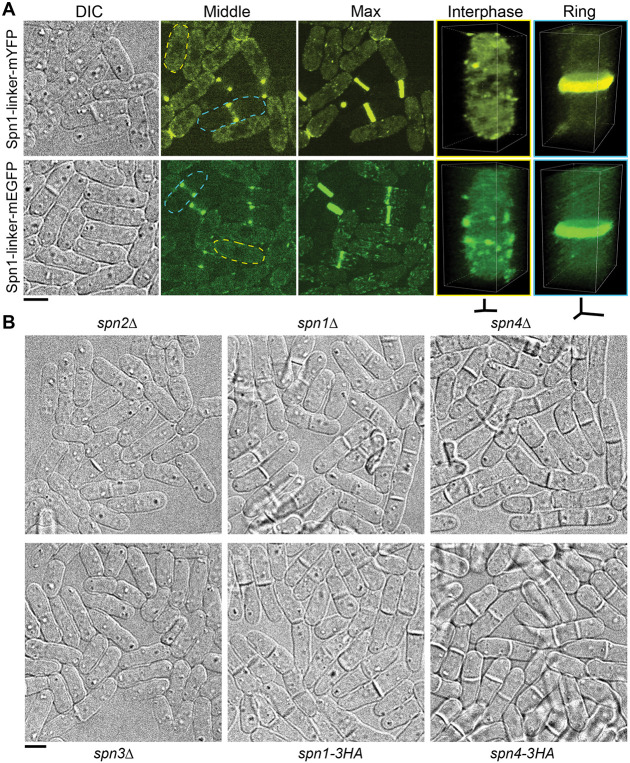
A flexible linker or smaller tag cannot make septin fusion proteins more functional. (A) Cell morphology and Spn1 localization shown for Spn1-linker-mYFP and Spn1-linker-mEGFP cells. Flexible linker sequence: ILGAPSGGGATAGAGGAGGPAGLI. DIC, fluorescence images at the middle focal plane and the maximal intensity projection of 25 slices with 0.3 μm spacing were shown. 3D volumetric projection of representative interphase and dividing cells are on the right. (B) DIC images showing the morphology of *spn2Δ, spn1Δ, spn4Δ, spn3Δ, spn1-3HA,* and *spn4-3HA* cells. Scale bars, 5 μm. Axial bars, 2 μm.

To assess if a flexible linker between a septin protein and epitope tag would render septin fusion proteins more functional, allowing them to polymerize normally, we inserted a long flexible linker between Spn1 and mYFP or mEGFP. The linker ILGAPSGGGATAGAGGAGGPAGLI was successfully used to construct functional EB1-GFP, which does not interfere with its binding with microtubules (Sandblad et al., 2006). Cells expressing Spn1-linker-mYFP or Spn1-linker-mEGFP had similar Spn1 localization as Spn1-mYFP and Spn1-mEGFP without the linker but had more Spn1 puncta or linear structures on the plasma membrane (Figure 3A). Thus, the linker does not improve the functionalities of tagged septins.

The tdTomato tag (476 aa) is roughly twice as big as GFP or YFP (239 aa); thus, we asked whether septins with smaller tags were more functional. To assess this, we tagged Spn1 and Spn4 with a much smaller 3HA tag at their C-termini (~39 aa in total that is used in yeast gene targeting; Bähler et al., 1998). Surprisingly, cells expressing Spn1-3HA or Spn4-3HA resembled *spn1Δ* and *spn4Δ* cells and were much more defective than *spn2Δ* or *spn3Δ* cells (Figure 3B and Table 1). Wild type (WT) *S. pombe* cells had ~11% of cells with a visible single septum and no cells with multi-septa in asynchronous population under our growth conditions (Table 1 and Figure 3B). Adding the 3HA tag increased the cells with a septum to ~67% and ~47% for Spn1-3HA and Spn4-3HA, respectively, and a small percentage of cells contained two or more septa (Table 1 and Figure 3B). In addition, more cells had a curved, instead of the normal straight morphology. This resulted in cells overlapping on slides during microscopy and led to an underestimation of the defects in quantification (Figure 3B). The high septation index and delay in daughter-cell separation of Spn1-3HA and Spn4-3HA cells resembled those of *spn1Δ* and *spn4Δ* cells (Table 1 and Figure 3B). Taken together, a flexible linker or much smaller tag such as 3HA does not enable tagged septins to be more functional.

### Tagged septins can alter each other’s localizations

**Figure 4:**
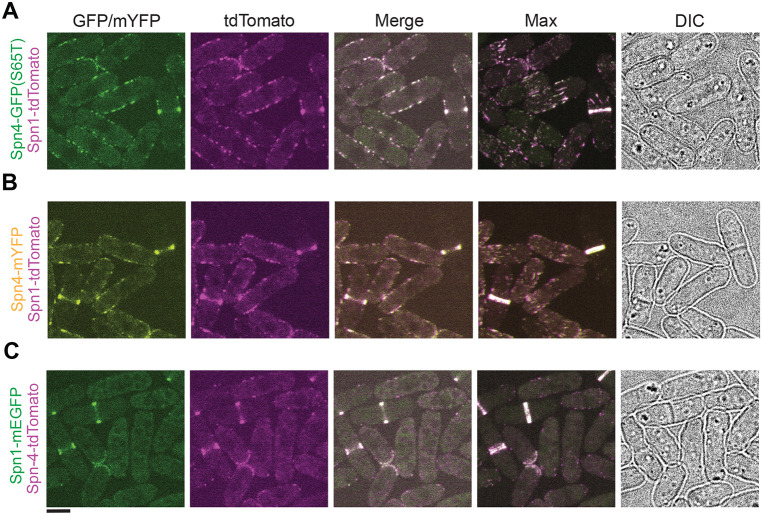
Tagged septins can affect each other’s localizations. Spn1 and Spn4 have a cooperative colocalization effect. Colocalization of Spn4-GFP(S65T) and Spn1-tdTomato (A), Spn4-mYFP and Spn1-tdTomato (B), and Spn1-mEGFP and Spn4-tdTomato (C). From left to right: GFP/YFP, tdTomato, and merged channels of the middle focal plane; the maximal intensity projection of 25 slices spaced at 0.3 μm for the merged channels; and DIC. Scale bar, 5 μm.

Next, we tested if septins colocalize in different structures on the plasma membrane in cells expressing one septin tagged with tdTomato, another with mEGFP, GFP(S65T), or mYFP (Figure 4). We found that the septins colocalized in all the structures including puncta, linear structures, and rings on the plasma membrane (Figure 4). In addition, it seemed that Spn1 was the dominant septin, since other septins followed the localization pattern of Spn1 regardless of whether it was in the puncta/linear structures or not during interphase (Figure 4, A-C). Strikingly, Spn4-tdTomato puncta in interphase mostly disappeared in cells coexpressing Spn1-mEGFP (Figure 4C). However, DIC images showed that cell morphology of all three strains had no obvious difference from WT (Figure 4 and Table 1). These results further show the caveats of studying septin localizations/interactions and the need for rigorous tests to determine their functionalities. Our data presented so far gives no convincing inferences to which septin localizations are native and which are artifacts. Thus, more sensitive tests are required to discern the functionality of tagged septins.

### Genetic interactions with the arrestin *art1Δ* reveal that tdTomato tagged septins are not functional

**Figure 5:**
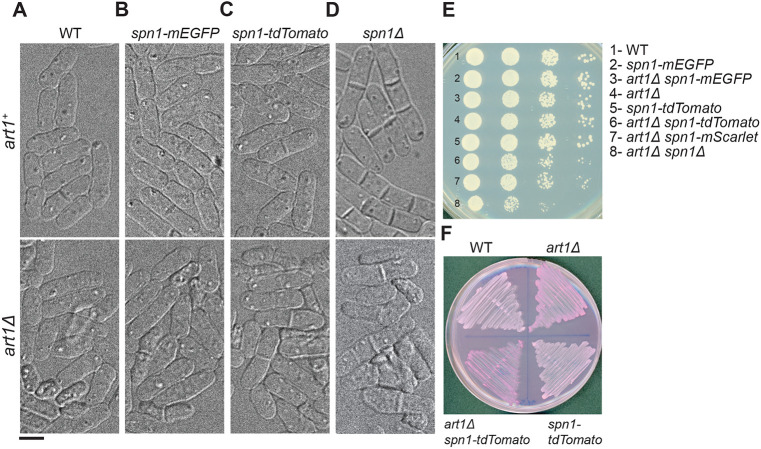
Synthetic genetic interactions between the arrestin *art1Δ* and tagged septins or *spn1Δ.* (A-D) Cell morphology under DIC of WT (A), *spn1-mEGFP* (B), *spn1-tdTomato* (C), and *spn1Δ* (D) cells in wild type *art1*^+^ (top) or *art1Δ* (bottom). Scale bar, 5 μm. (E) Serial dilutions (10x) to test growth of the eight indicated strains. Cells were grown exponentially in YE5S for ~36 h before spotting on YE5S plates. The plates were grown for ~48 h at 36°C (E), 32°C, and 28°C. The growth patterns were the same at the three temperatures. (F) Synthetic genetic interactions between *art1Δ* and *spn1-tdTomato.* Cells were grown at 36°C for 48h on YE5S plates + Phloxin B, which stains lysed or dead cells red. Darker red color indicates more cell lysis.

The cell morphology of all the strains expressing tagged septins examined so far (except 3HA) resembled WT (Figures 1-4). Spn1-mEGFP, Spn1-tdTomato, Spn4-mYFP, and Spn4-tdTomato all showed no significant difference in either cell lysis or septation index (Table 1 and Figure 5, A-C, upper panels). This indicated that quantification is not sufficient to distinguish which fluorescently tagged septins retain their native localization and which exhibit altered localization. Moreover, growth and color on plates with Phloxin B from 20 to 36°C were similar for these tagged septin strains. Thus, it was necessary to use more sensitive genetic interactions to test the functionalities of tagged septins. Art1 is an arrestin-like protein that functions in a parallel pathway with septins to control cytokinesis and cell integrity (Wu et al., 2010; Davidson et al., 2015). Double mutants of *art1* and septin caused significant cell lysis during daughter-cell separation (Wu et al., 2010). To elucidate the effects on cell health more precisely, we crossed the fluorescently tagged septin strains with *art1Δ*. We hypothesized that strains expressing functional septins will display the same phenotype as the *art1Δ* mutant. In contrast, cells expressing less functional septin fusion proteins will show more perturbed phenotypes with *art1Δ*. We found that *art1Δ spn1-mEGFP* and *art1Δ spn4-mYFP* cells exhibited the same phenotype as *art1Δ* (Table 1 and Figure 5, A and B). All had <23% of lysed cells and with a similar septation index (<13%). In contrast, *art1Δ spn1-tdTomato* and *art1Δ spn4-tdTomato* strains had ≥35% of cells lysed. This was similar to *art1Δ spn1Δ* and *art1Δ spn4Δ* cells, which had 49% and 30% of cells lysed, respectively (Table 1 and Figure 5, C and D). Cell lysis in the double deletion strains was underestimated due to extensive cell crowding and overlapping. In addition, the septation index was much higher in *art1Δ spn1Δ* and *art1Δ spn4Δ* cells (Table 1). Furthermore, serial dilutions and growth on plates with Phloxin B confirmed our results (Figure 5, E and F). In the serial dilutions, *art1Δ spn1-tdTomato* grew less than both *art1Δ spn1-mEGFP* and *art1Δ* (Figure 5E), and *art1Δ spn1-tdTomato* cells had more cell lysis than either single mutant, which was evident from the darker red color on Phloxin B plates grown at 36°C (Figure 5F). In addition, cells expressing Spn1-mScarlet, Spn1-mRFP1, and Spn4-mScarlet had higher septation index (Table 1), and the localization pattern of Spn1-mCherry was similar to that of Spn1-tdTomato (unpublished data). Collectively, these data indicate that mEGFP and mYFP tagged septins are more functional than septins tagged with tdTomato or other red fluorescent proteins.

### Septin localizations to the plasma membrane puncta or linear structures are independent of the anillin Mid2

**Figure 6:**
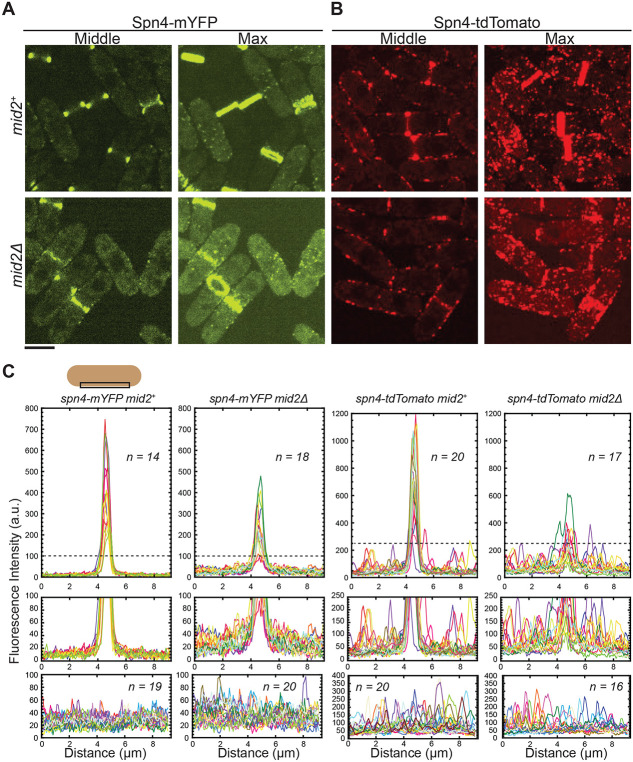
Localization of Spn4 to the division site but not to the interphase membrane puncta or linear structures depends on the anillin Mid2. Localization of Spn4-mYFP (A) and Spn4-tdTomato (B) in *mid2*^+^ (top) and *mid2Δ* (bottom) cells. Images of the middle focal plane and maximal intensity projection of 25 slices with 0.3 μm spacing are shown. Scale bar, 5 μm. (C) Plots of fluorescence intensity on the plasma membrane measured using the middle focal plane along the cell-long axis (9.2 μm long centered at the cell middle within a width of 0.92 μm) for cells with the septin rings (top and middle) and cells in interphase (bottom). The schematic shows the membrane region that was quantified. The dashed line (in top graph) marks the portion of the graph enlarged below (middle graph) to view the signal outside the division site with higher resolution.

The anillin Mid2 colocalizes with septins at the division site and is important for septin assembly and organization (Berlin et al., 2003; Tasto et al., 2003; Wu et al., 2010; Arbizzani et al., 2022). We tested if the formation of septin puncta outside the division site on the plasma membrane depends on Mid2. In *mid2*^+^ cells, Spn4-mYFP concentrated to the division site as rings during cytokinesis, and the signals in the cytoplasm and on the plasma membrane outside the division site were very faint (Figure 6, A and C). During interphase, Spn4-mYFP diffused throughout the cytoplasm and rarely in the puncta on the plasma membrane (Figure 6, A and C). This observation is consistent with Figure 2A. In *mid2Δ* cells, Spn4-mYFP remained diffused in the cytoplasm during interphase as in *mid2*^+^ cells. However, Spn4-mYFP intensity in the rings decreased in *mid2Δ* cells during cytokinesis (Figure 6, A and C), consistent with previous reports (Berlin et al., 2003; Tasto et al., 2003; Wu et al., 2010; Arbizzani et al., 2022). Spn4-mYFP signal increased in cytoplasm and the plasma membrane outside the division site (Figure 6, A and C). The division site signal of Spn4-tdTomato also dramaticallydecreased during cytokinesis in *mid2Δ* cells; however, Spn4-tdTomato puncta and linear structures persisted on the plasma membrane during interphase and outside the division site during cytokinesis (Figure 6, B and C). Thus, it can be concluded that the cortical localization of Spn4-tdTomato outside the division site, unlike the normal septin localization in the rings, is Mid2 independent.

### The localization of NDR kinase Sid2 to the division site is delayed in septin mutants

It has been proposed that septin puncta and linear structures on the plasma membrane in cells expressing Spn1-tdTomato or Spn4-tdTomato serve as a diffusion barrier that affects the localizations and functions of the NDR kinase Sid2, the Rho GAPs Rga6 and Rga4, and active Cdc42 (Zheng et al., 2018; Zheng et al., 2022; McDuffie et al., 2024). Because we have found that tdTomato tagged septins are less functional than mEGFP or mYFP tagged septins, we reexamined the relationship between Sid2 and septins.

**Figure 7:**
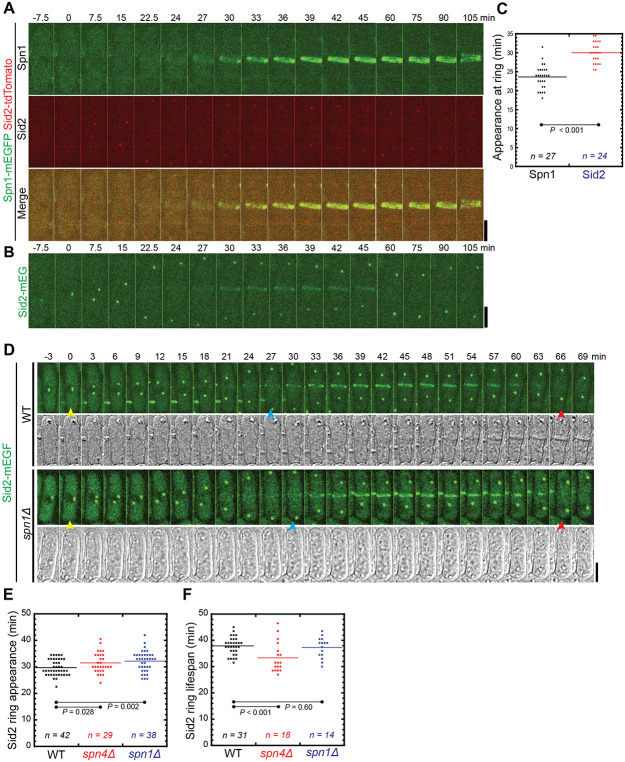
Sid2 localization at the division site in WT and septin mutants. (A-C) Time courses (A and B) and quantification (C) of Spn1 and Sid2 appearance at the division site. Time courses of maximal intensity projection (9 slices with 0.9 μm spacing) of Spn1 (A) and Sid2 (B). Cells expressing Spn1-mEGFP Sid2-tdTomato or Sid2-mEGFP alone were mixed at ~1:1 ratio and imaged on the same slide. SPB separation as marked by Sid2 is defined as time 0. Because Sid2-tdTomato signal is weak, Sid2-mEGFP was used to quantify Sid2 appearance in (C). (D-F) Sid2 appearance and duration at the division site in WT and septin mutants. (D) Time courses of maximal intensity projection (13 slices with 0.5 μm spacing) of Sid2-mEGFP in WT and *spn1Δ* cells. Yellow arrows: SPB separation at the onset of mitosis; Blue arrows: Sid2 arrival at division site; and Red arrows: Sid2 disappearance of the division site. Scale bars, 5 μm. (E and F) Quantification of (E) Sid2-mEGFP ring appearance at the division site (SPB separation set as time 0) and (F) the lifespan of Sid2-mEGFP (from Sid2 ring appearance to its departure) in WT, *spn1Δ,* and *spn4Δ* cells. P values were calculated by Student’s t-test.

Sid2 is the most downstream kinase in the SIN (septation initiation network) pathway, whose homologs function in the equivalent MEN pathway in budding yeast and HIPPO pathway in animal cells including humans (McCollum and Gould, 2001; Yoshida et al., 2002; Hergovich and Hemmings, 2012; Heallen et al., 2013; Hsu and Weiss, 2013; Foltman and Sanchez-Diaz, 2017; Sinha et al., 2022). Sid2 localizes to the spindle pole body (SPB) through the cell cycle and localizes to the division site during cytokinesis (Sparks et al., 1999). A previously proposed model suggests that septin-tdTomato puncta on the plasma membrane inhibit Sid2’s localization to the division site; once the septin rings are formed, they help to maintain SIN signaling (Zheng et al., 2018). Sid2 appearance at the division site was reported significantly advanced, and Sid2 duration at the cleavage furrow was significantly shortened in *spn1Δ* cells compared to WT (Zheng et al., 2018). Using time-lapse confocal microscopy, we found that both Spn1-mEGFP and Spn4-mYFP appeared in the rings at the division site ~6 minutes earlier than Sid2-mEGFP in WT cells (Figure 7, A-C). In *spn1Δ* and *spn4Δ* cells, Sid2 arrived at the division site at 32.2 ± 3.8 and 31.5 ± 3.8 min after SPB separation, respectively, which were delayed compared to 29.7 ± 3.0 in the WT cells (Figure 7, D and E; p = 0.002 and 0.028). Additionally, we observed that Sid2 persisted at the division site similarly in WT and *spn1Δ* cells, whereas Sid2 disappeared earlier in *spn4Δ* cells (Figure 7F). In WT and *spn1Δ* cells, Sid2 departed from the division site at ~38 min and ~37 min after its appearance, respectively; while Sid2 stayed for ~33 min at the division site in *spn4Δ* cells (Fig 7F). Together, the findings that septins appear at the division site earlier than Sid2 in WT cells and that Sid2 appearance is delayed in septin deletion mutants do not support the model that septins serve as a diffusion barrier to prevent Sid2 localization to the division site.

## DISCUSSION

In this study, we investigate the discrepancy over the localizations and functions of septins during interphase in fission yeast as detailed in the Introduction (Berlin et al., 2003; Tasto et al., 2003; An et al., 2004; Zheng et al., 2018; Zheng et al., 2022; McDuffie et al., 2024). Though all the tagged septin proteins are expressed at their native chromosomal loci and under the control of their native promotors, Spn1 and Spn4 tagged with tdTomato or mScarlet are not as functional as their mEGFP or mYFP tagged counterparts. Additionally, small tags such as 3HA completely disrupt septin functions because cells expressing Spn1-3HA or Spn4-3HA resemble septin deletion mutants. Our data suggest that the reported septin puncta or linear filaments/structures on the plasma membrane during interphase are caused by non-functional Spn1-tdTomato or Spn4-tdTomato, and that they may not serve as interphase diffusion barriers for proteins involved in cytokinesis and cell polarity.

### Epitope tags may induce septin mislocalization and dysfunction

By assessing septin fusion proteins in live fission yeast cells, we elucidated the necessity of using caution when interpreting the localizations and functions of septins. We find that different epitope tags can perturb both the functions and localizations of septins. Several lines of evidence indicate that mEGFP and mYFP-tagged septins maintain normal function and localization at the division site, whereas 3HA, tdTomato, and other red fluorescence protein-tagged septins suffer from partial to complete loss of functions. First, cells expressing Spn1 or Spn4 tagged with mEGFP or mYFP resemble WT in morphology, septation index, growth at different temperatures, and they have no synthetic genetic interactions with the arrestin *art1Δ* (Figures 1, 2, 5 and Table 1). Second, cells expressing 3HA-tagged Spn1 and Spn4 resemble *spn1Δ* or *spn4Δ* in percent of cells with defects in septum formation or delay in daughter-cell separation, and cell lysis (Figure 5 and Table 1). Although 3HA is a small tag relative to fluorescent proteins, it entirely disrupts septin functions. Third, cells expressing Spn1 or Spn4 with the tdTomato tag resemble WT cells in their morphology and septation index. However, in double mutants with *art1Δ,* these cells have >50% increase in cell lysis compared to *art1Δ* cells. Because Spn1-tdTomato and Spn4-tdTomato spread all over the plasma membrane even during cytokinesis, the levels of septins at the division site are much lower than cells with mEGFP or mYFP tagged septins (Figures 1, 2, and 6). The reduced septin level cannot sustain septin function in cytokinesis without Art1. Thus, tdTomato and 3HA tags lead to partial or complete loss of septin functions.

We also do not think that septin puncta or linear filaments/structures across the plasma membrane outside the division site in cells with tdTomato or other tags serve as diffusion barriers. First, Sid2 appearance at the division site is later in septin deletion mutants rather than earlier compared to in WT cells (Figure 7, D and E). In addition, septins appear at the division site several minutes earlier than Sid2 (Figure 7, A-C), which is not consistent with the idea that septins inhibit Sid2 localization. However, Sid2 is less abundant than septins at the division site (Figure 7, A and B). Thus, we cannot rule out that Sid2 appears at the division site several minutes earlier than what we determined. However, it is unlikely that Sid2 appears at the division site earlier than septins because Sid2-tdTomato signal is very weak but its appearance time at the division site is similar to that of Sid2-mEGFP. Second, cells expressing Spn1-mEGFP Spn4-tdTomato and Spn4-mYFP Spn1-tdTomato have very similar morphology, septation index, and very low cell-lysis percentage (Table 1) despite Spn1 and Spn4 localizations being dramatically different during interphase in these cells (Figure 4). Third, the anillin Mid2 plays an important role in septin localization and organization at the division site during cytokinesis (Berlin et al., 2003; Tasto et al., 2003; Wu et al., 2010; Arbizzani et al., 2022). However, Spn4-tdTomato localization during interphase does not depend on Mid2 (Figure 6). Thus, it is more likely that septin puncta or linear structures during interphase in cells with tdTomato or other tags have no significant functional roles. To our knowledge, no report has shown that septins localize across the entire plasma membrane in both mother and daughter cells during interphase in budding yeast.

### Why various epitope tags have drastically different effects on septin localizations and functions

It is likely that different tags interfere with or affect the interactions between septin subunits and the septin-plasma membrane differently. Septins form palindromic, oligomeric structures when they bind to one another at both the NC face and the G face (Sirajuddin et al., 2007). It is likely that 3HA and tdTomato tags, but not mEGFP or mYFP, interferes with the NC face since all the tags are added C-terminally. It has also been shown that Spn1-YFP, but not Spn1-mYFP (monomeric YFP with A206K) leads to formation of straight, long septin bundle in fission yeast (Davidson et al., 2016). This corroborates that septin localizations and assembly are susceptible to epitope taggings.

Septins bind with the plasma membrane by interacting with anionic lipids like PI(4,5)P2 and phosphatidylserine and then polymerize into filaments (Zhang et al., 1999; Bridges et al., 2014). Septins also preferentially recognize and interact with plasma membrane with micron-scale curvature (Bridges et al., 2016). In the rod-shaped fission yeast cells, the membrane curvatures at the cleavage furrow, cell sides, and cell tips are quite different. The localizations and functions of septins are also regulated by phosphorylation and ubiquitination (Johnson and Blobel, 1999; Vargas-Muñiz et al., 2016). It is possible that epitope tags such as tdTomato triggers a conformational change in septin subunits that leads to a loss in septins’ curvature or lipid preference. It is clear that tdTomato compromises the membrane specificity of both Spn1 and Spn4. It is also possible that the epitope tags induce the same structural changes that mimic a posttranslational modification. These possibilities may explain the mixed results that we observed when expressing two differently tagged septins at once (Figure 4). Whatever the reasons, it seems that different epitope tags affect these determinants of septin localizations differently. Future studies are needed to determine how epitope tags interfere with septins’ membrane specificity.

### Functionality tests of tagged septins in other systems

Septins play essential roles in cytokinesis, cell polarization, sporulation, and many other cellular processes across diverse cell types by forming higher order structures (Garcia et al., 2011). In many cell types, it is technically challenging to test the functionality of widely used epitope-tagged septins, even when these septins are expressed at their endogenous levels. Most known functionality tests have been done in budding yeast by rescue mutant phenotype or by observing cell morphology (Chang, 1999; Johnson and Blobel, 1999). The budding yeast has at least two advantages in evaluating functionalities of tagged septins compared to fission yeast and all other cell types. First, most septins are essential in budding yeast and multiple temperature-sensitive alleles are available. Nonfunctional tagged septins do not survive or would have shown obvious phenotypes. Second, the pioneering septin localization studies were done using septin-specific antibodies before the discovery of GFP and other fluorescent proteins. Thus, nonfunctionally tagged septin proteins are less likely to appear in literature. When GFP fusions of CDC3, CDC10, CDC11, and CDC12 were first utilized in budding yeast, there were varying levels of assessments of their functionality. Studies utilizing CDC10-GFP and CDC3-GFP made no direct statements about the functionality of these fusion proteins (Cid et al., 1998; Moffat and Andrews, 2004). A study utilizing all four fusion proteins claimed that all four were fully functional (Takahashi et al., 1999). However, Chang et al. found that the addition of GFP causes a decrease in CDC12 functionality, and CDC12-GFP cannot completely restore *cdc12* loss-of-function mutant phenotype (Chang, 1999). Unfortunately, functionality tests of tagged septins have not been rigorously performed in many other systems.

Our study reinforces a vital need for rigorous functionality tests of epitope tagged proteins. It has been previously shown that fluorescent tags can alter the localization and binding interactions of proteins (Snapp, 2009; Fatti et al., 2025). In the context of condensate formation, tagging Dhh1, an mRNA degradation factor enriched in P-bodies, with GFP or mCherry2 inhibits its normal condensation ability (Fatti et al., 2025). Moreover, tagged G-actin monomers with GFP or other tags impedes actin’s incorporation into linear actin filaments nucleated by formins (Doyle and Botstein, 1996; Wu and Pollard, 2005; Chen et al., 2012). Many cytokinesis proteins are not functional when tagged at one of the termini (Wu et al., 2003). Because epitope tagged proteins are widely used for cellular and biochemical studies of septins and essentially all other proteins, cautions must be used when interpreting protein localizations, interactions, and functions, especially for proteins that polymerize into heteromeric high-order structures, such as septins.

## MATERIALS AND METHODS

### Yeast strains and genetic methods

The fission yeast *S. pombe* strains used in this study are listed in Table 2. The yeast strains were woken up from −80°C stocks onto YE5S plates and grown at 25°C for 2 to 3 days before experiments. Gene targeting was performed using the standard PCR-based homologous recombination method (Bähler et al., 1998). To construct double mutants, crosses and tetrad dissection were carried out according to the standard genetic method (Moreno et al., 1991). Briefly, fresh growing cells of opposite mating type (*h*^+^ or *h*^−^) were mixed in sterile water on a SPA5S plate and incubated at 25° for ~48 h. The cross was streaked atop a YE5S plate for tetrad dissection using a Nikon 50i dissection microscope, and the plate was incubated at 25°C for 5 to 7 days. The colonies of sufficient sizes were picked up using sterile toothpicks onto a new YE5S plate and grown for one or two more days. Then they were replica plated onto selection YE5S plates with antibiotics to identify the double mutants.

### Confocal microscopy and data analyses

Confocal microscopy was performed as before with some modifications to minimize perturbation of septin localizations (Davidson et al., 2016; Singh et al., 2024). Briefly, fresh cells from the strains that were woken up on a YE5S plate were inoculated into a triple baffled 50 ml glass flask with ~10 ml of YE5S liquid medium. The cells were grown in the dark in a waterbath shaker with a speed of 150 rpm at 25°C for 36 to 48 h. Cells were diluted twice a day with new YE5S medium to ensure that the strains were growing at log phase with OD_595_ <0.5. Before microscopy, 0.9 ml of culture was mixed with 0.1 ml of 50 μM n-propyl gallate made in YE5S medium and incubated for 2 min to protect cells from free radicals during microscopy. We collected the cells by centrifugation at 3,000 rpm for 30 s. The extra supernatant was discarded, the cell pellet was resuspended in the remaining ~10 to 20 μl medium. ~3 to 5 μl of the resuspended cells were then spotted onto a gelatin pad (20% gelatin in YE5S liquid medium containing n-propyl gallate at a final concentration of 5 μM) on a microscope slide. The cells were covered with a No. 1.5 cover slip (22 x 22 mm) and sealed with VALAP just before imaging. Cells were imaged with a Nikon CSU-W1 SoRa spinning disk confocal microscope with Hamamatsu ORCA Quest qCMOS camera C15550 on Nikon Eclipse Ti2 microscope with Plan Apo λD 100x/1.45 NA oil objective. Yeast mutants or strains with various fluorophores were imaged. For strains with a mYFP tag, the 514 nm laser was set at 5-20% power; for GFP-based tags, the 488 nm laser at 25-50%; for tdTomato, mScarlet, or other red fluorescent proteins, the 561 nm laser at 20-50%. For all strains, DIC images were taken to examine cell morphology, but DIC was somehow compromised by the Piezo used for the confocal imaging. Images and fluorescence intensity were analyzed or measured using NIS Elements and Fiji software (Singh et al., 2024).

## Figures and Tables

**Table 1:** Septation index and cell lysis in tagged septin or septin mutant strains.

Strain	Number of cells scored	% of cells with the indicated number of septa^*a,b*^			% of lysed or shrunken cells
		0	1	≥2	
WT	633	88.6	11.4	0.0	0.47
*art1Δ* ^*c*^	1,371	87.4	12.6	0.0	22.9
*spn1-3HA*	680	31.5	66.7	1.8	0.15
*spn1-mEGFP*	705	91.6	8.40	0.0	0.43
*art1Δ spn1-mEGFP*	840	92.6	7.40	0.0	22.9
*spn1-tdTomato*	669	86.3	13.7	0.0	0.90
*art1Δ spn1-tdTomato*	951	84.8	15.1	0.2	36.5
*spn1-mScarlet*	707	76.9	23.1	0.0	0.14
*art1Δ spn1-mScarlet*	842	91.0	9.00	0.0	24.5
*spn1Δ*	473	34.0	63.3	2.8	0.42
*art1Δ spn1Δ*	743	60.5	36.3	3.2	48.9
*spn4-3HA* ^*c*^	1,848	51.9	47.0	1.2	0.87
*spn4-mYFP*	684	91.1	8.90	0.0	0.15
*art1Δ spn4-mYFP*	595	93.7	6.30	0.0	15.1
*spn4-tdTomato*	459	86.1	13.9	0.0	0.00
*art1Δ spn4-tdTomato*	774	83.8	16.0	0.2	34.5
*spn4-mScarlet*	568	67.1	32.6	0.4	1.58
*art1Δ spn4-mScarlet* ^*c*^	1,360	69.7	29.7	0.6	24.4
*spn4Δ* ^*c*^	1,152	28.8	67.0	3.6	1.56
*art1Δ spn4Δ*	525	42.4	53.8	3.8	29.9
*spn4-mYFP spn1-tdTomato*	699	88.7	11.3	0.0	1.14
*spn4-tdTomato spn1-mEGFP*	942	85.8	14.1	0.1	0.53

Cells were grown in log phase in YE5S liquid medium for ~36 h at 25°C before imaging.

a The lysed or shrunken cells were excluded from the septation index calculation.

b Septa were counted using DIC images, which resulted in an underestimate because the emerging septum may not be easily visible.

c For these strains, results were combined from two separate imaging sessions.

**TABLE 2:** *S. pombe* strains utilized in this study.

Strain name	Genotype	Figure panel
Figure 1		
JW1092	*h*^−^ *spn1-mYFP-kanMX6 ade6-M210 leu1-32 ura4-D18*	A
JW1091	*h*^−^ *spn1-mEGFP-kanMX6 ade6-M210 leu1-32 ura4-D18*	B
JW1345	*h^−^ spn1-tdTomato-natMX6 ade6-M210 leu1-32 ura4-D18*	C
Figure 2		
JW9844	*h*^−^ *spn4-mYFP-kanMX6 ade6-M210 leu1-32 ura4-D18*	A
JW8589	*spn4-GFP(S65T)-kanMX6 ade6 leu1-32 ura4-D18*	B
JW8843	*h^−^ spn4-tdTomato-natMX6 ade6-M210 leu1-32 ura4-D18*	C
Figure 3		
JW1934	*h^−^ spn1-linker-mYFP-kanMX6 ade6-M210 ura4-D18 leu1-32*	A
JW1935	*h*^−^ *spn1-linker-mEGFP-kanMX6 ade6-M210 ura4-D18 leu1-32*	A
JW9732	h- spn2-Δ1::hphMX6 ade6-210 ura4-D18 leu1-32	B
JW1507	spn1-Δ2::kanMX6 ade6-M210 leu1-32 ura4-D18	B
JW9724	h- spn4-Δ2::hphMX6 ade6-210 ura4-D18 leu1-32	B
JW709	h- spn3-Δ2::kanMX6 ade6-M210 leu1-32 ura4-D18	B
JW250	h- spn1-3HA-kanMX6	B
JW184	*h*^−^ *spn4-3HA-kanMX6*	B
Figure 4		
JW10244	*spn4-GFP(S65T)-kanMX6 spn1-tdTomato-natMX6 ade6 leu1-32 ura4-D18*	A
JW10242	*spn4-mYFP-kanMX6 spn1-tdTomato-natMX6 ade6-M210 leu1-32 ura4-D18*	B
JW10245	*spn1-mEGFP-kanMX6 spn4-tdTomato-natMX6 ade6-M210 leu1-32 ura4-D18*	C
Figure 5		
JW81	*h*^−^ *ade6-M210 leu1-32 ura4-D18*	A, E, F
JW3710	*h*^+^ *art1Δ::kanMX6 ade6-210 leu1-32 ura4-D18*	A, E, F
JW1091	*h*^−^ *spn1-mEGFP-kanMX6 ade6-M210 leu1-32 ura4-D18*	B, E
JW10259	*art1Δ::kanMX6 spn1-mEGFP-kanMX6 ade6-210 leu1-32 ura4-D18*	B, E
JW1345	*h^−^ spn1-tdTomato-natMX6 ade6-M210 leu1-32 ura4-D18*	C, E, F
JW10260	*art1Δ::kanMX6 spn1-tdTomato-natMX6 ade6-210 leu1-32 ura4-D18*	C, E, F
JW1507	*spn1-Δ2::kanMX6 ade6-M210 leu1-32 ura4-D18*	D
JW10272	*art1Δ::kanMX6 spn1-Δ2::kanMX6 ade6-210 leu1-32 ura4-D18*	D, E
Figure 6		
JW1171	*h*^+^ *spn4-mYFP-kanMX6 ade6-M210 leu1-32 ura4-D18*	A, C
JW10297	*mid2-Δ1::kanMX6 spn4-mYFP-kanMX6 ade6-M210 leu1-32 ura4-D18*	A, C
JW10256	*h^+^ spn4-tdTomato-natMX6 ade6-M210 leu1-32 ura4-D18*	B, C
JW10300	*mid2-Δ1::kanMX6 spn4-tdTomato-natMX6 ade6-M210 leu1-32 ura4-D18*	B, C
Figure 7		
JW10304	*spn1-mEGFP-kanMX6 sid2-tdTomato-kanMX6 ade6-M210 leu1-32 ura4-D18*	A, C
JW10125	*h^−^ sid2-mEGFP-hphMX6 ade6-210 ura4-D18 leu1-32*	B, C
JW10327	*sid2-mEGFP-hphMX6 spn1-Δ2::kanMX6 ade6-210leu1-32 ura4-D18*	D, E, F
JW10308	*h^+^ sid2-mEGFP-hphMX6 ade6-210 ura4-D18 leu1-32*	D, E, F
JW10326	*sid2-mEGFP-hphMX6 spn4-Δ2::hphMX6 ade6-210 leu1-32 ura4-D18*	E, F
Table 1		
JW81	*h*^−^ *ade6-M210 leu1-32 ura4-D18*	
JW3710	*h^+^ art1Δ::kanMX6 ade6-210 leu1-32 ura4-D18*	
JW250	*h*^−^ *spn1-3HA-kanMX6*	
JW1091	*h*^−^ *spn1-mEGFP-kanMX6 ade6-M210 leu1-32 ura4-D18*	
JW10259	*art1Δ::kanMX6 spn1-mEGFP- kanMX6 ade6-210 leu1-32 ura4-D18*	
JW1345	*h^−^ spn1-tdTomato-natMX6 ade6-M210 leu1-32 ura4-D18*	
JW10260	*art1Δ::kanMX6 spn1-tdTomato-natMX6 ade6-210 leu1-32 ura4-D18*	
JW8275	*h^−^ spn1-mScarlet-I-hphMX6 ade6-210 ura4-D18 leu1-32*	
JW10261	*art1Δ::kanMX6 spn1-mScarlet-I-hphMX6 ade6-210 leu1-32 ura4-D18*	
JW1507	*spn1-Δ2::kanMX6 ade6-M210 leu1-32 ura4-D18*	
JW10272	*art1Δ::kanMX6 spn1-Δ2::kanMX6 ade6-210 leu1-32 ura4-D18*	
JW184	*h*^−^ *spn4-3HA-kanMX6*	
JW9844	*h*^−^ *spn4-mYFP-kanMX6 ade6-M210 leu1-32 ura4-D18*	
JW10263	*art1Δ::kanMX6 spn4-mYFP-kanMX6 ade6210 leu1-32 ura4-D18*	
JW8843	*h^−^ spn4-tdTomato-natMX6 ade6-M210 leu1-32 ura4-D18*	
JW10293	*art1Δ::kanMX6 spn4-tdTomato-natMX6 ade6-210 leu1-32 ura4-D18*	
JW8844	*h^−^ spn4-mScarlet-I-hphMX6 ade6-M210 leu1-32 ura4-D18*	
JW10294	*art1Δ::kanMX6 spn4-mScarlet-I-hphMX6 ade6-210 leu1-32 ura4-D18*	
JW9724	*h*^−^ *spn4-Δ2::hphMX6 ade6-210 ura4-D18 leu1-32*	
JW10264	*art1Δ::kanMX6 spn4-Δ2::hphMX6 ade6-210 leu1-32 ura4-D18*	
JW10242	*spn4-mYFP-kanMX6 spn1-tdTomato-natMX6 ade6-M210 leu1-32 ura4-D18*	
JW10245	*spn1-mEGFP-kanMX6 spn4-tdTomato-natMX6 ade6-M210 leu1-32 ura4-D18*	

## SIGNIFICANCE STATEMENT

- Fission yeast septins Spn1 and Spn4, expressed under their native promotors, have dramatically distinct localizations on the plasma membrane with different fluorescent tags.
- By assessing cell morphology, cell-division defects, cell integrity, and genetic interactions between tagged septins and other mutations, we find that septins are not functional when tagged with tdTomato or 3HA but are functional when tagged with mEGFP or mYFP.
- Our data highlights the need for rigorous functional tests of tagged septins and caution when interpreting localization/function data because septin polymers are susceptible to perturbations. Our results also raise awareness of studying other proteins because epitope tagging is widely used.

## Abbreviations:

DIC: differential interference contrast
SIN: septation initiation network
SPB: spindle pole body
tdTomato: tandem dimer Tomato
WT: wild type
