# Supplementary material for: Localization and function of septins are susceptible to epitope tagging
Source: bioRxiv. 2025 Sep 24:2025.05.13.653749. Originally published 2025 May 14. Preprint. [Version 2] doi: 10.1101/2025.05.13.653749 (PMC12132285; doi:10.1101/2025.05.13.653749)
Supplement: 1 [file NIHPP2025.05.13.653749v2-supplement-1.pdf]

bioRxiv preprint doi: https://doi.org/10.1101/2025.05.13.653749; this version posted September 24, 2025. The copyright holder for this preprint (which was not certified by peer review) is the author/funder, who has granted bioRxiv a license to display the preprint in perpetuity. It is made available under aCC-BY 4.0 International license.

SUPPLEMENTAL MATERIALS

Molecular Biology of the Cell

Localization and function of septins are susceptible to epitope tagging

Jack R. Gregory, Ian Mikale A. Llaneza, Aysha H. Osmani, Haley E. Gosselin, S. Amirreza Sabzian, and Jian-Qiu Wu

Movie Legend

Movie 1: Comparison of the localization of Spn1-tdTomato and Spn1-linker-tdTomato. Spn1-tdTomato (JW1345) and Spn1-linker-tdTomato (JW10434) cells were grown in log phase at 25°C for ~36 h before imaging. The movie shows the maximal intensity projection of 11 focal planes with 0.7 µm spacing at each time point. The images were taken every 1 min for 30 min. 7 frames per second.

bioRxiv preprint doi: https://doi.org/10.1101/2025.05.13.653749; this version posted September 24, 2025. The copyright holder for this preprint (which was not certified by peer review) is the author/funder, who has granted bioRxiv a license to display the preprint in perpetuity. It is made available under aCC-BY 4.0 International license.

Supplemental Table S1: *S. pombe* strains utilized in this study.

Strain name	Genotype	Figure/Table
**Figure 1**		
JW1092	*h⁻ spn1-mYFP-kanMX6 ade6-M210 leu1-32 ura4-D18*	A
JW1091	*h⁻ spn1-mEGFP-kanMX6 ade6-M210 leu1-32 ura4-D18*	B
JW1345	*h⁻ spn1-tdTomato-natMX6 ade6-M210 leu1-32 ura4-D18*	C
**Figure 2**		
JW9844	*h⁻ spn4-mYFP-kanMX6 ade6-M210 leu1-32 ura4-D18*	A
JW8589	*spn4-GFP(S65T)-kanMX6 ade6 leu1-32 ura4-D18*	B
JW8843	*h⁻ spn4-tdTomato-natMX6 ade6-M210 leu1-32 ura4-D18*	C
JW310	*h- 41nmt1-GFP(S65T)-spn4+ ade6-M210 leu1-32 ura4-D18*	D
**Figure 3**		
JW9732	*h- spn2Δ::hphMX6 ade6-210 ura4-D18 leu1-32*	A
JW1507	*spn1Δ::kanMX6 ade6-M210 leu1-32 ura4-D18*	A
JW9724	*h- spn4Δ::hphMX6 ade6-210 ura4-D18 leu1-32*	A
JW709	*h- spn3Δ::kanMX6 ade6-M210 leu1-32 ura4-D18*	A
JW250	*h- spn1-3HA-kanMX6*	A
JW184	*h- spn4-3HA-kanMX6*	A
JW10453	*spn1-3HA-kanMX6 spn4-mYFP-kanMX6 ade6-M210 leu1-32 ura4-D18*	B
JW10454	*spn4-3HA-kanMX6 spn1-mEGFP-kanMX6 ade6-M210 leu1-32 ura4-D18*	B
**Figure 4**		
JW10244	*spn4-GFP(S65T)-kanMX6 spn1-tdTomato-natMX6 ade6 leu1-32 ura4-D18*	A
JW10242	*spn4-mYFP-kanMX6 spn1-tdTomato-natMX6 ade6-M210 leu1-32 ura4-D18*	B
JW10245	*spn1-mEGFP-kanMX6 spn4-tdTomato-natMX6 ade6-M210 leu1-32 ura4-D18*	C
**Figure 5**		
JW1171	*h⁺ spn4-mYFP-kanMX6 ade6-M210 leu1-32 ura4-D18*	A, C
JW10297	*mid2Δ::kanMX6 spn4-mYFP-kanMX6 ade6-M210 leu1-32 ura4-D18*	A, C
JW10256	*h⁺ spn4-tdTomato-natMX6 ade6-M210 leu1-32 ura4-D18*	B, C
JW10300	*mid2Δ::kanMX6 spn4-tdTomato-natMX6 ade6-M210 leu1-32 ura4-D18*	B, C
JW10376	*spn4-tdTomato-natMX6 mid2-mEGFP-kanMX6 ade6-M210 leu1-32 ura4-D18*	D
**Figure 6**		
JW81	*h⁻ ade6-M210 leu1-32 ura4-D18*	A, E, F
JW3710	*h⁺ art1Δ::kanMX6 ade6-210 leu1-32 ura4-D18*	A, E, F
JW1091	*h⁻ spn1-mEGFP-kanMX6 ade6-M210 leu1-32 ura4-D18*	B, E
JW10259	*art1Δ::kanMX6 spn1-mEGFP-kanMX6 ade6-210 leu1-32 ura4-D18*	B, E, G
JW1345	*h⁻ spn1-tdTomato-natMX6 ade6-M210 leu1-32 ura4-D18*	C, E, F
JW10260	*art1Δ::kanMX6 spn1-tdTomato-natMX6 ade6-210 leu1-32 ura4-D18*	C, E, F, G
JW10261	*art1Δ::kanMX6 spn1-mScarlet-I-hphMX6 ade6-210 leu1-32 ura4-D18*	C
JW1507	*spn1Δ::kanMX6 ade6-M210 leu1-32 ura4-D18*	D
JW10272	*art1Δ::kanMX6 spn1Δ::kanMX6 ade6-210 leu1-32 ura4-D18*	D, E

bioRxiv preprint doi: https://doi.org/10.1101/2025.05.13.653749; this version posted September 24, 2025. The copyright holder for this preprint (which was not certified by peer review) is the author/funder, who has granted bioRxiv a license to display the preprint in perpetuity. It is made available under aCC-BY 4.0 International license.

(Continued)

Strain name	Genotype	Figure panel
JW8843	*h⁻ spn4-tdTomato-natMX6 ade6-M210 leu1-32 ura4-D18*	F
JW10293	*art1Δ::kanMX6 spn4-tdTomato-natMX6 ade6-210 leu1-32 ura4-D18*	F, G
JW10428	*art1Δ::kanMX6 spn1-mYFP-kanMX6 ade6-210 leu1-32 ura4-D18*	G
JW10429	*art1Δ::kanMX6 spn1-YFP-kanMX6 ade6-210 leu1-32 ura4-D18*	G
JW10422	*art1Δ::kanMX6 spn1-mEGFP-kanMX6 spn4-tdTomato-natMX6 ade6-210 leu1-32 ura4-D18*	G
**Figure 7**		
JW10304	*spn1-mEGFP-kanMX6 sid2-tdTomato-kanMX6 ade6-M210 leu1-32 ura4-D18*	A, C
JW10125	*h⁻ sid2-mEGFP-hphMX6 ade6-210 ura4-D18 leu1-32*	B, C
JW10327	*sid2-mEGFP-hphMX6 spn1Δ::kanMX6 ade6-210leu1-32 ura4-D18*	D, E, F
JW10308	*h⁺ sid2-mEGFP-hphMX6 ade6-210 ura4-D18 leu1-32*	D, E, F
JW10326	*sid2-mEGFP-hphMX6 spn4Δ::hphMX6 ade6-210 leu1-32 ura4-D18*	E, F
**Supplemental Figure S1**		
JW309	*h⁻ 3nmt1-GFP(S65T)-spn4+ ade6-M210 leu1-32 ura4-D18*	A
JW310	*h⁻ 41nmt1-GFP(S65T)-spn4+ ade6-M210 leu1-32 ura4-D18*	B
JW311	*h⁻ 81nmt1-GFP(S65T)-spn4+ ade6-M210 leu1-32 ura4-D18*	C
**Supplemental Figure S2**		
JW1934	*h⁻ spn1-linker-mYFP-kanMX6 ade6-M210 ura4-D18 leu1-32*	A
JW1935	*h⁻ spn1-linker-mEGFP-kanMX6 ade6-M210 ura4-D18 leu1-32*	B
JW10434	*h⁻ spn1-linker-tdTomato-kanMX6 ade6-210 ura4-D18 leu1-32*	C
JW1345	*h⁻ spn1-tdTomato-natMX6 ade6-M210 leu1-32 ura4-D18*	D
**Supplemental Figure S3**		
JW399	*h- cdc4-s16-F3 leu1-32 ura4-D18 ade6-M210*	A, B
JW10416	*cdc4-s16-F3 spn4-mYFP-kanMX6 ade6-M210 leu1-32 ura4-D18*	A
JW10414	*cdc4-s16-F3 spn4-tdTomato-natMX6 ade6-M210 leu1-32 ura4-D18*	A
JW10415	*cdc4-s16-F3 spn1-mEGFP-kanMX6 ade6-M210 leu1-32 ura4-D18*	A, B
JW10427	*cdc4-s16-F3 spn1-tdTomato-natMX6 ade6-M210 leu1-32 ura4-D18*	A, B
JW81	*h⁻ ade6-M210 leu1-32 ura4-D18*	B
**Supplemental Figure S4**		
JW10293	*art1Δ::kanMX6 spn4-tdTomato-natMX6 ade6-210 leu1-32 ura4-D18*	A
JW10422	*art1Δ::kanMX6 spn1-mEGFP-kanMX6 spn4-tdTomato-natMX6 ade6-210 leu1-32 ura4-D18*	A
JW10428	*art1Δ::kanMX6 spn1-mYFP-kanMX6 ade6-210 leu1-32 ura4-D18*	B, C
JW10429	*art1Δ::kanMX6 spn1-YFP-kanMX6 ade6-210 leu1-32 ura4-D18*	B, C
**Table 1**		
JW81	*h⁻ ade6-M210 leu1-32 ura4-D18*	
JW3710	*h⁺ art1Δ::kanMX6 ade6-210 leu1-32 ura4-D18*	
JW250	*h⁻ spn1-3HA-kanMX6*	

bioRxiv preprint doi: https://doi.org/10.1101/2025.05.13.653749; this version posted September 24, 2025. The copyright holder for this preprint (which was not certified by peer review) is the author/funder, who has granted bioRxiv a license to display the preprint in perpetuity. It is made available under aCC-BY 4.0 International license.

(Continued)

Strain name	Genotype
JW1091	*h⁻ spn1-mEGFP-kanMX6 ade6-M210 leu1-32 ura4-D18*
JW10259	*art1Δ::kanMX6 spn1-mEGFP- kanMX6 ade6-210 leu1-32 ura4-D18*
JW1345	*h⁻ spn1-tdTomato-natMX6 ade6-M210 leu1-32 ura4-D18*
JW10260	*art1Δ::kanMX6 spn1-tdTomato-natMX6 ade6-210 leu1-32 ura4-D18*
JW10434	*h- spn1-linker-tdTomato-kanMX6 ade6-210 ura4-D18 leu1-32*
JW10452	*art1Δ::kanMX6 spn1-linker-tdTomato-kanMX6 ade6-210 ura4-D18 leu1-32*
JW8275	*h- spn1-mScarlet-I-hphMX6 ade6-210 ura4-D18 leu1-32*
JW10261	*art1Δ::kanMX6 spn1-mScarlet-I-hphMX6 ade6-210 leu1-32 ura4-D18*
JW1507	*spn1Δ::kanMX6 ade6-M210 leu1-32 ura4-D18*
JW10272	*art1Δ::kanMX6 spn1Δ::kanMX6 ade6-210 leu1-32 ura4-D18*
JW184	*h⁻ spn4-3HA-kanMX6*
JW9844	*h⁻ spn4-mYFP-kanMX6 ade6-M210 leu1-32 ura4-D18*
JW10263	*art1Δ::kanMX6 spn4-mYFP-kanMX6 ade6210 leu1-32 ura4-D18*
JW8843	*h⁻ spn4-tdTomato-natMX6 ade6-M210 leu1-32 ura4-D18*
JW10293	*art1Δ::kanMX6 spn4-tdTomato-natMX6 ade6-210 leu1-32 ura4-D18*
JW8844	*h⁻ spn4-mScarlet-I-hphMX6 ade6-M210 leu1-32 ura4-D18*
JW10294	*art1Δ::kanMX6 spn4-mScarlet-I-hphMX6 ade6-210 leu1-32 ura4-D18*
JW9724	*h⁻ spn4Δ::hphMX6 ade6-210 ura4-D18 leu1-32*
JW10264	*art1Δ::kanMX6 spn4Δ::hphMX6 ade6-210 leu1-32 ura4-D18*
JW10242	*spn4-mYFP-kanMX6 spn1-tdTomato-natMX6 ade6-M210 leu1-32 ura4-D18*
JW10245	*spn1-mEGFP-kanMX6 spn4-tdTomato-natMX6 ade6-M210 leu1-32 ura4-D18*
JW10422	*art1Δ::kanMX6 spn1-mEGFP-kanMX6 spn4-tdTomato-natMX6 ade6-210 leu1-32 ura4-D18*
JW10428	*art1Δ::kanMX6 spn1-mYFP-kanMX6 ade6-210 leu1-32 ura4-D18*
JW10429	*art1Δ::kanMX6 spn1-YFP-kanMX6 ade6-210 leu1-32 ura4-D18*
**Table 2**	
JW399	*h- cdc4-s16-F3 leu1-32 ura4-D18 ade6-M210*
JW10415	*cdc4-s16-F3 spn1-mEGFP-kanMX6 ade6-M210 leu1-32 ura4-D18*
JW10427	*cdc4-s16-F3 spn1-tdTomato-natMX6 ade6-M210 leu1-32 ura4-D18*
JW10416	*cdc4-s16-F3 spn4-mYFP-kanMX6 ade6-M210 leu1-32 ura4-D18*
JW10414	*cdc4-s16-F3 spn4-tdTomato-natMX6 ade6-M210 leu1-32 ura4-D18*
JW10452	*cdc4-s16-F3 spn1-linker-tdTomato-kanMX6 ade6-210? his7-366? ura4-D18 leu1-32*

bioRxiv preprint doi: https://doi.org/10.1101/2025.05.13.653749; this version posted September 24, 2025. The copyright holder for this preprint (which was not certified by peer review) is the author/funder, who has granted bioRxiv a license to display the preprint in perpetuity. It is made available under aCC-BY 4.0 International license.

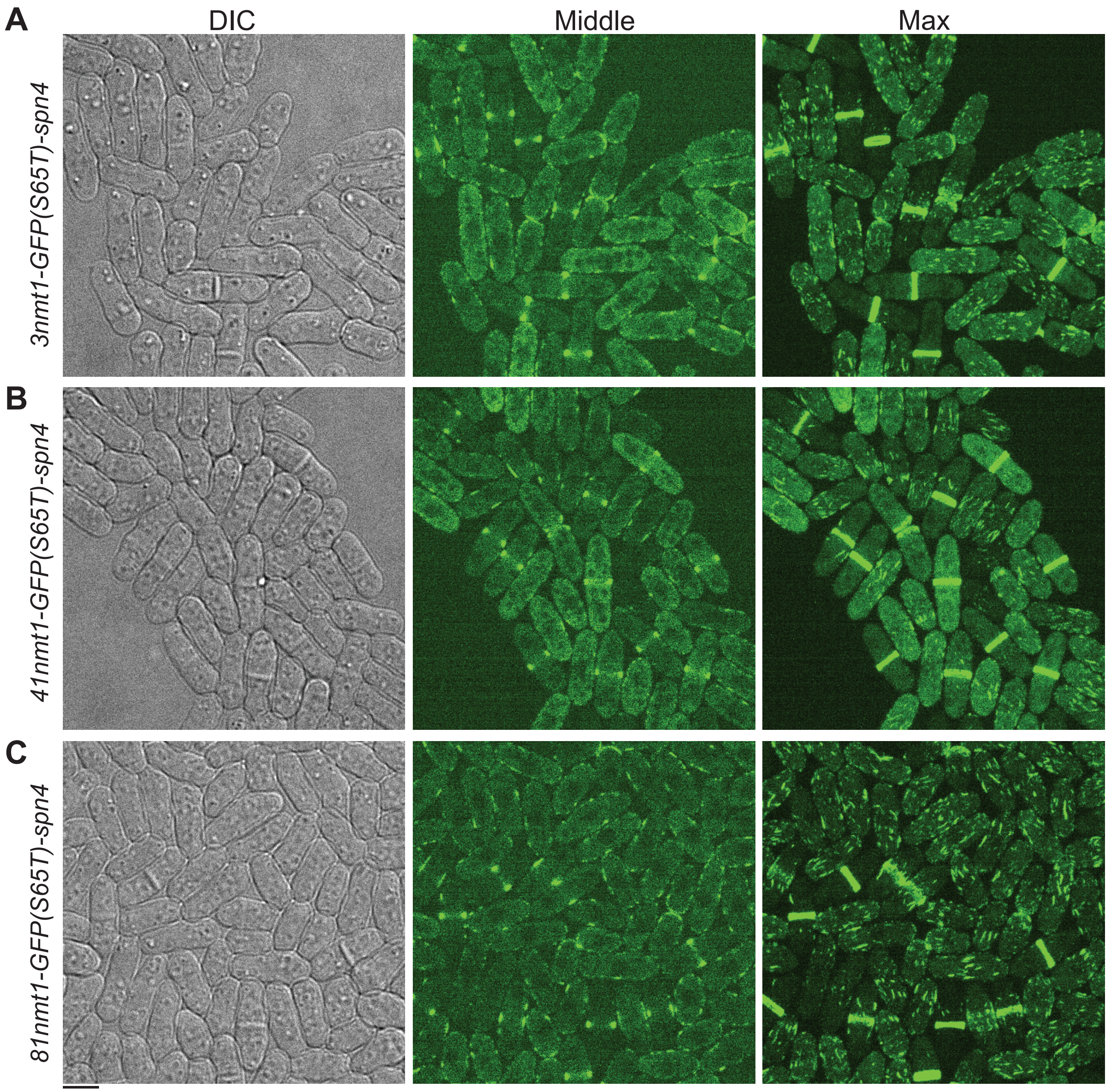

Supplemental Figure S1. Cells expressing GFP(S65T)-Spn4 at various levels (controlled by the *nmt1* promoters with different strengths) show similar phenotype and localizations. *3nmt1-GFP(S65T)-spn4* cells in YE5S (A), *41nmt1-GFP(S65T)-spn4* cells in EMM5S (B), and *81nmt1-GFP(S65T)-spn4* cells in EMM5S (C). Cells were grown in the indicated liquid media for 20-22 h before imaging. DIC, fluorescence images at the middle focal plane, and the maximal intensity projection of 25 slices with 0.3 µm spacing are shown. Scale bar, 5 µm.

Supplemental Figure S1

bioRxiv preprint doi: https://doi.org/10.1101/2025.05.13.653749; this version posted September 24, 2025. The copyright holder for this preprint (which was not certified by peer review) is the author/funder, who has granted bioRxiv a license to display the preprint in perpetuity. It is made available under aCC-BY 4.0 International license.

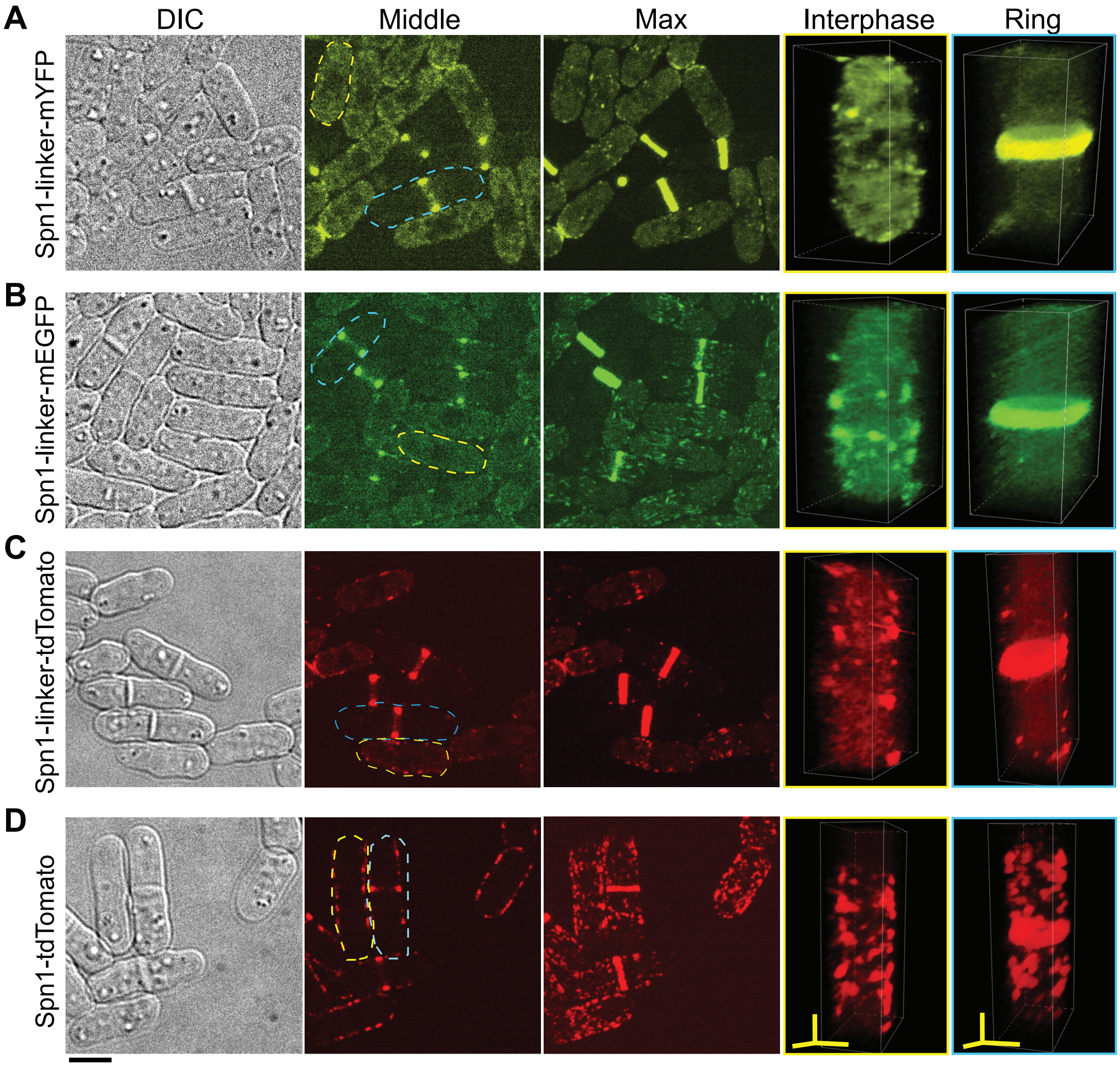

Supplemental Figure S2. A flexible linker has varying effects on septin Spn1 localizations. Cell morphology and Spn1 localization of cells expressing Spn1-linker-mYFP (A), Spn1-linker-mEGFP (B), Spn1-linker-tdTomato (C), and Spn1-tdTomato cells (D). DIC, fluorescence images at the middle focal plane and the maximal intensity projection are shown. 37 slices with 0.2 µm spacing (A and B) and 19 slices with 0.4 µm spacing (C and D). 3D volumetric projection of representative interphase and dividing cells (Ring) are on the right. Scale bar, 5 µm. Axial bars, 2 µm.

Supplemental Figure S2

bioRxiv preprint doi: https://doi.org/10.1101/2025.05.13.653749; this version posted September 24, 2025. The copyright holder for this preprint (which was not certified by peer review) is the author/funder, who has granted bioRxiv a license to display the preprint in perpetuity. It is made available under aCC-BY 4.0 International license.

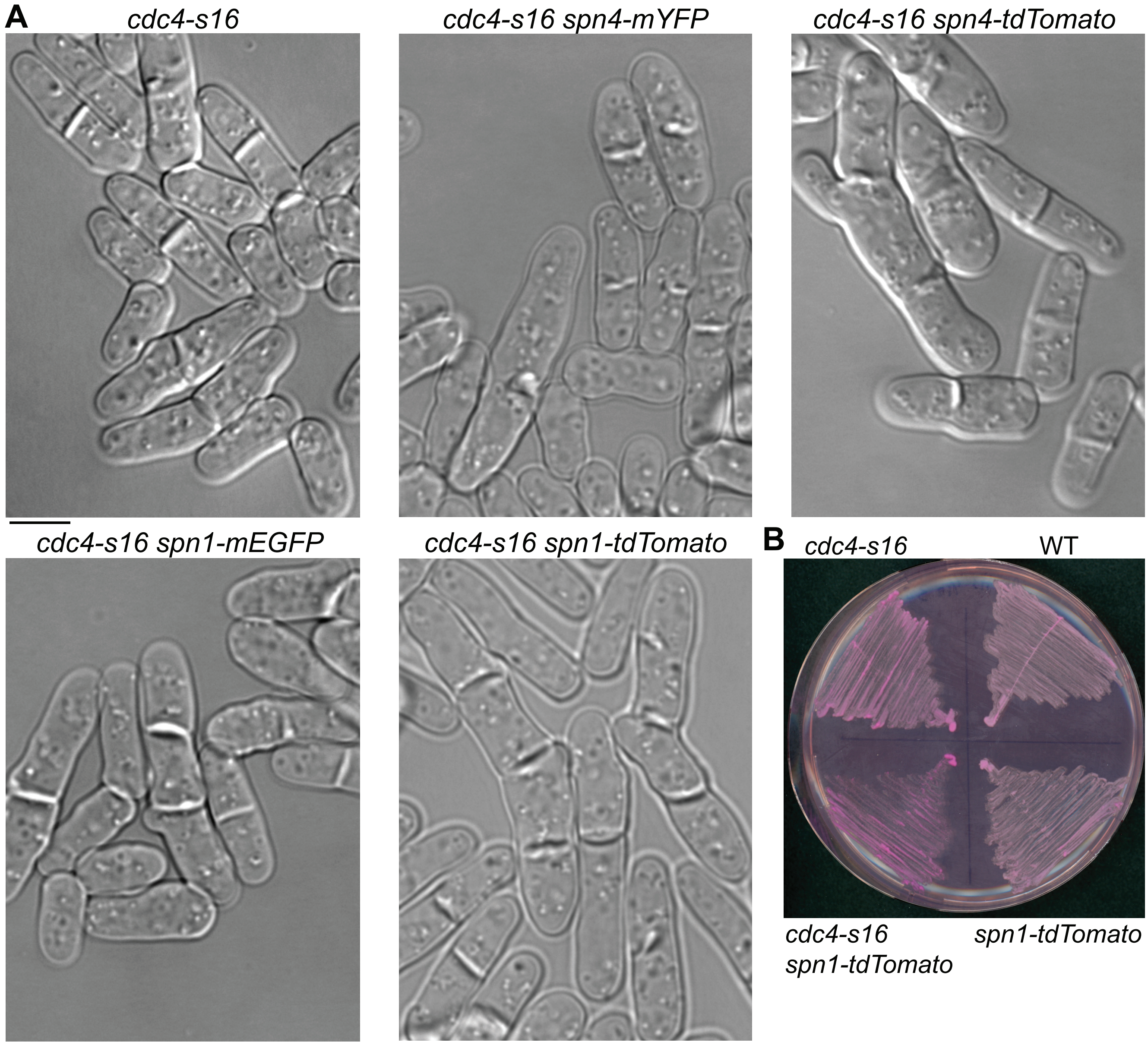

Supplemental Figure S3: Synthetic genetic interactions between cold-sensitive *cdc4-s16* mutant and tagged septins. (A) Representative DIC images of the indicated strains. Cells were grown in log phase in YE5S liquid medium for ~36 h at 32°C then shifted to 25°C for 4 h before imaging. Scale bar, 5 µm. (B) Growth of the indicated strains on YE5S + Phloxin B plate at 32°C for ~29 h.

Supplemental Figure S3

bioRxiv preprint doi: https://doi.org/10.1101/2025.05.13.653749; this version posted September 24, 2025. The copyright holder for this preprint (which was not certified by peer review) is the author/funder, who has granted bioRxiv a license to display the preprint in perpetuity. It is made available under aCC-BY 4.0 International license.

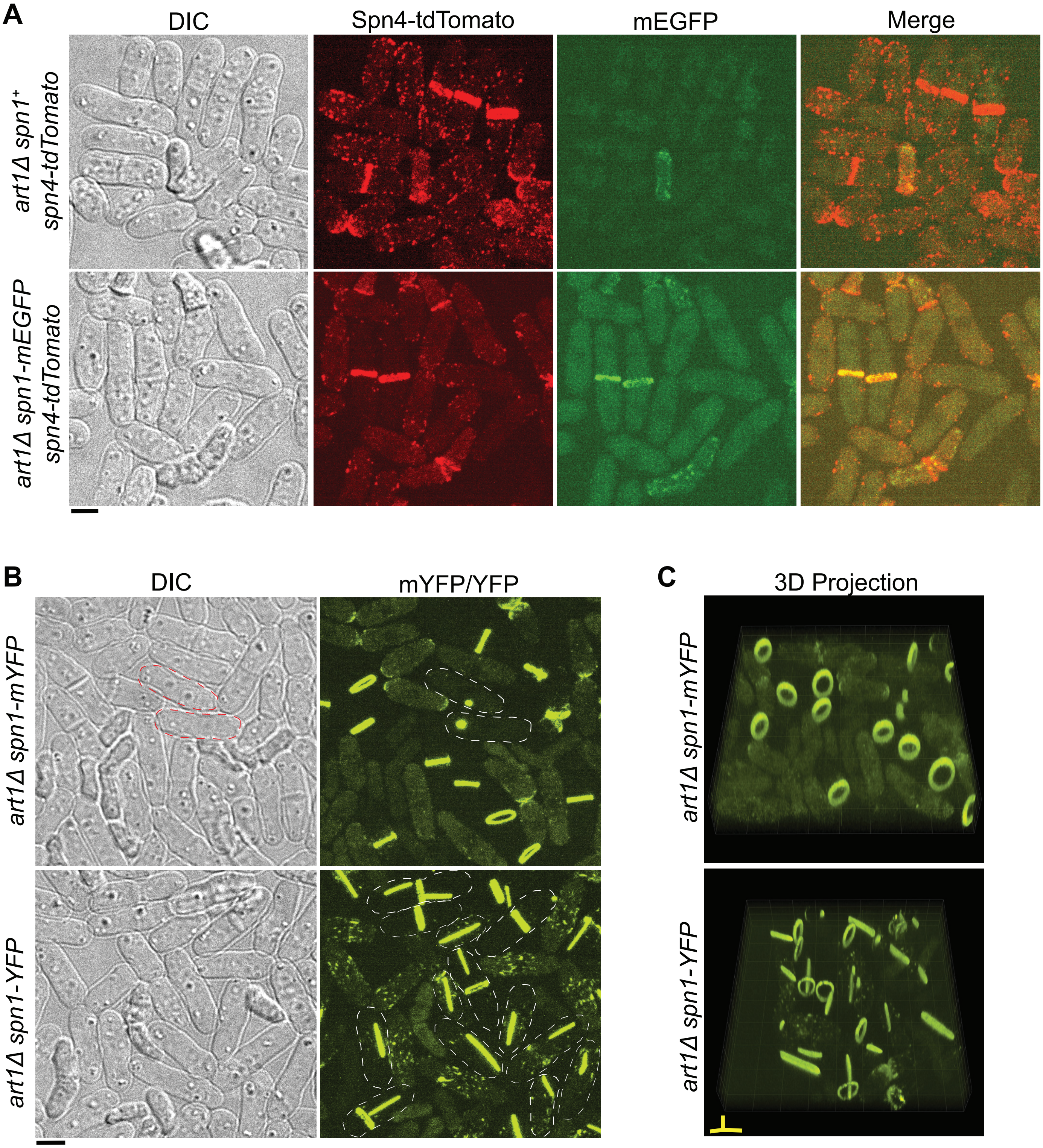

Supplemental Figure S4. Localizations of the septins Spn1 and Spn4 in *art1Δ* cells. (A) Localizations of Spn4-tdTomato or Spn4-tdTomato Spn1-mEGFP in *art1Δ* cells. Cell morphology and maximal intensity projections of 25 slices with 0.3 µm spacing are shown. Top, untagged Spn1; bottom, Spn1-mEGFP. (B and C) Spn1-YFP but not Spn1-mYFP forms septin bars or spirals in the cytoplasm besides the septin rings at the division site in *art1Δ* cells. (B) DIC and maximal intensity projections of 19 slices with 0.4 µm spacing are shown. (C) 3D projections of fluorescence images. Representative cells are outlined using dashed lines. Spn1-mYFP appears as a discrete spot in a small fraction of cells (outlined). Scale bar, 5 µm.

Supplemental Figure S4

bioRxiv preprint doi: https://doi.org/10.1101/2025.05.13.653749; this version posted September 24, 2025. The copyright holder for this preprint (which was not certified by peer review) is the author/funder, who has granted bioRxiv a license to display the preprint in perpetuity. It is made available under aCC-BY 4.0 International license.

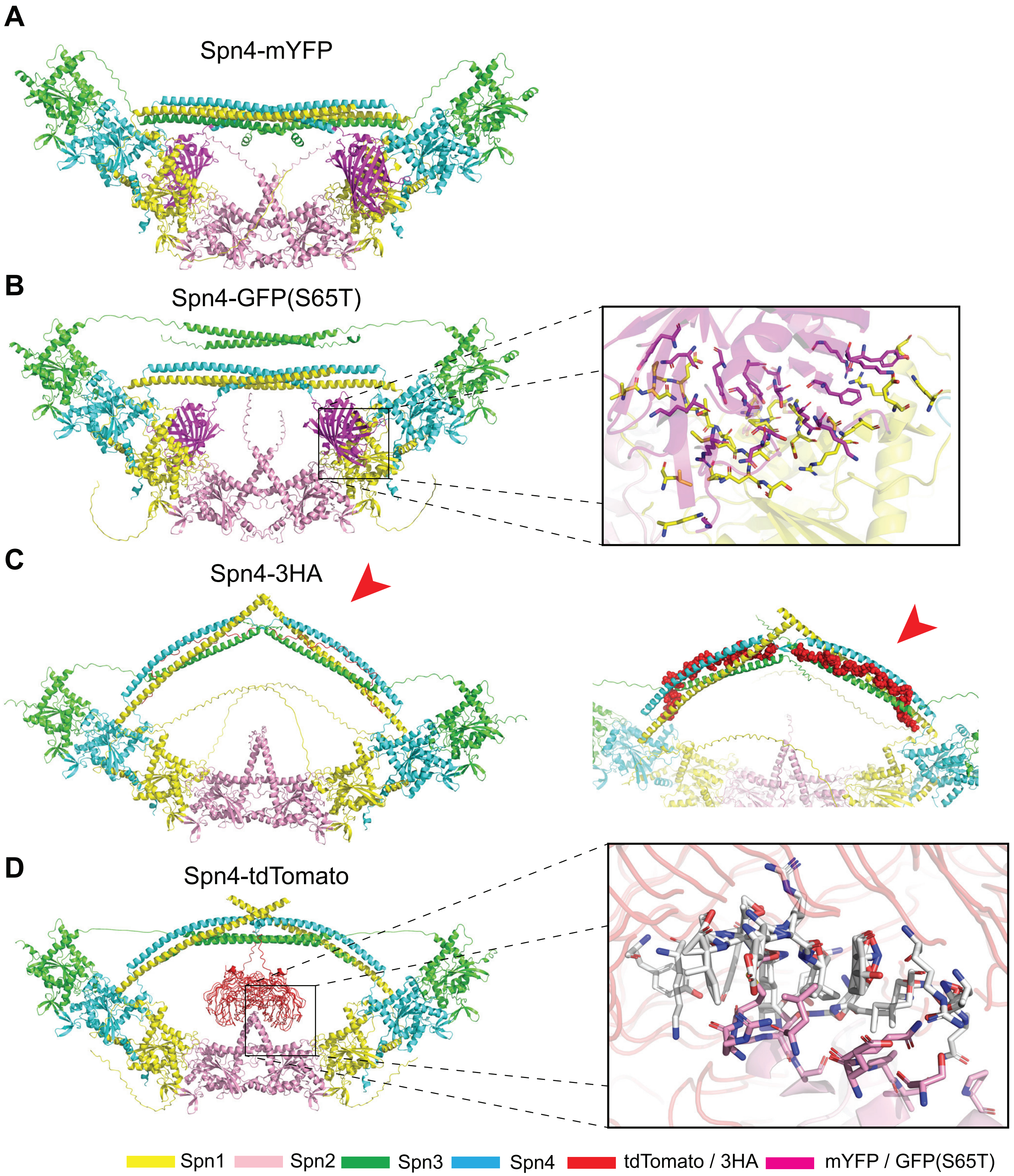

Supplemental Figure S5: Predicted structures of the septin octameric filament with epitope-tagged Spn4 by AlphaFold3. (A-D) Septins Spn1-4 are differentiated by the color as labeled. Two subunits of each septin were used to construct the octameric filament. tdTomato and 3HA are colored in BR9 (red), and GFP(S65T) and mYFP are colored in magenta. Red arrowheads indicate the 3HA interaction with the bundled coiled coils. (B and D) Box projections show interacting residues between (B) GFP(S65T) (magenta) and Spn1 (yellow) or (D) tdTomato (white) and Spn2 (pink) within 3.5Å, indicative of Van der Waals forces represented as sticks. (A) Spn4-mYFP model, pTM = 0.45. (B) Spn4-GFP(S65T) model, pTM = 0.45. (C) Spn4-3HA, pTM = 0.49. (D) Spn4-tdTomato model, pTM = 0.47.

Supplemental Figure S5

bioRxiv preprint doi: https://doi.org/10.1101/2025.05.13.653749; this version posted September 24, 2025. The copyright holder for this preprint (which was not certified by peer review) is the author/funder, who has granted bioRxiv a license to display the preprint in perpetuity. It is made available under aCC-BY 4.0 International license.

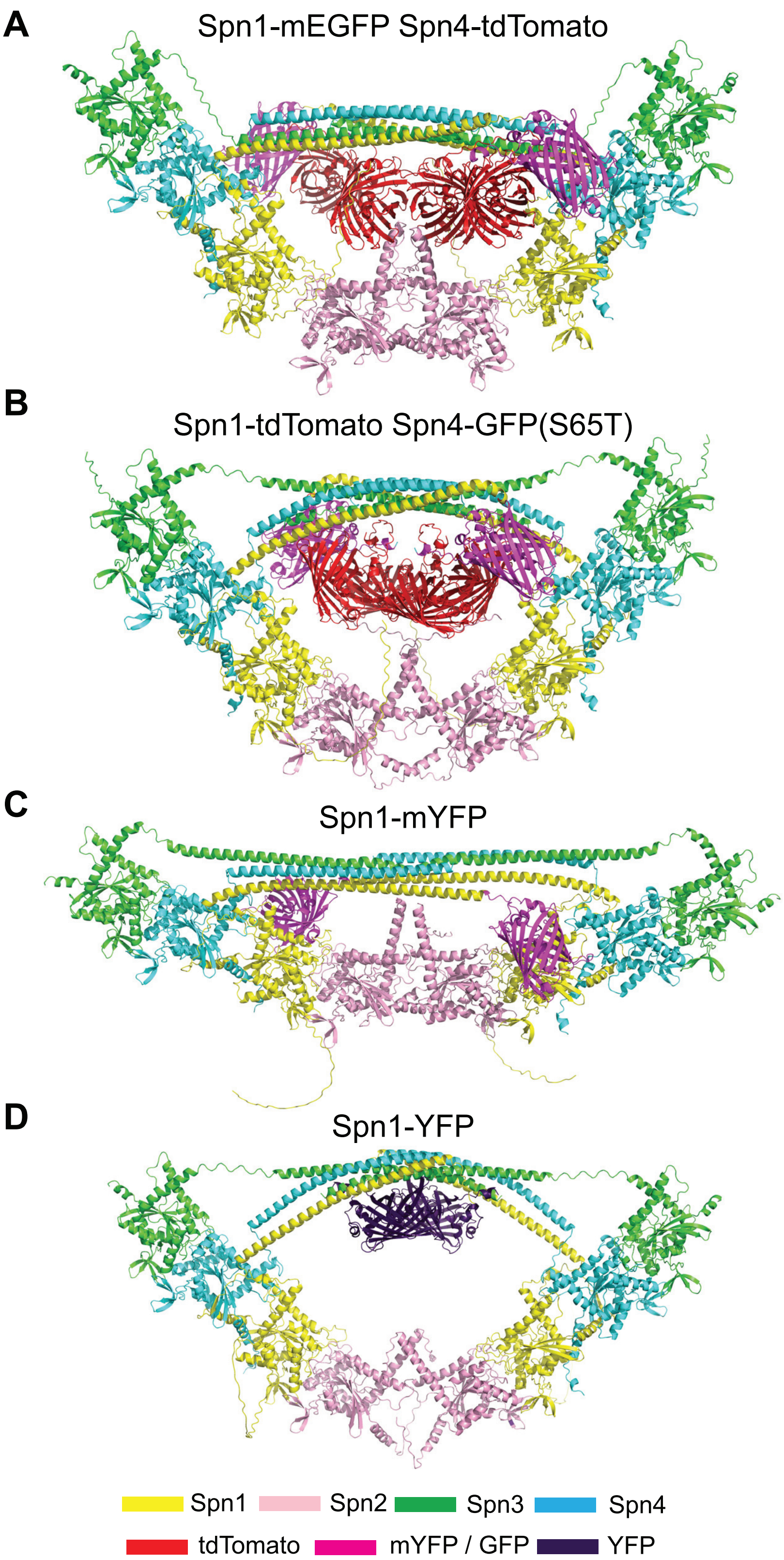

Supplemental Figure S6: Predicted structures of the septin octameric filament of monomeric and dimeric YFP-tagged Spn1 and the cooperative colocalization effect by AlphaFold3. (A-D) Septins Spn1-4 are differentiated by the color as labeled. Two subunits of each septin were used to construct the octameric filament. tdTomato is colored in BR9 (red), GFP/mYFP is colored in magenta, and the dimeric YFP is colored in francium (dark purple). (A) Spn1-mEGFP Spn4-tdTomato model, pTM = 0.41. (B) Spn1-tdTomato Spn4-GFP(S65T) model, pTM = 0.42. (C) Spn1-mYFP model, pTM = 0.49. (D) Spn1-YFP model. pTM = 0.45.

Supplemental Figure S6
